# Management Practices of Cats Owned by Faculty, Staff, and Students at Two Midwest Veterinary Schools

**DOI:** 10.1155/2016/7108374

**Published:** 2016-12-20

**Authors:** Judith L. Stella, Candace C. Croney

**Affiliations:** Center for Animal Welfare Science, Purdue University, West Lafayette, IN, USA

## Abstract

Understanding cat owners' housing, care, and management practices is important for promoting cat welfare. A survey study was conducted on the housing and management practices used for cats by students, faculty, and staff of The Ohio State University and Purdue University veterinary colleges. Subjects were 138 cat-owner dyads. Most cats (74%) were housed strictly indoors in keeping with common US veterinary recommendations. However, many did not implement best practices outlined for behavior and other welfare needs of indoor cats. The percentage of respondents placing resources where cats could be disrupted while using them was 31%, 53%, and 30% for resting areas, food/water dishes, and litter boxes, respectively. Many cats were not provided a litter box in a private area (35%), in multiple areas of the house (51%), or that was regularly washed (73%). Horizontal scratching opportunities were not provided to 38% of cats; 32% were not provided toys that mimic prey and 91% of cats were fed a diet consisting of >75% dry food. These findings suggest a need for more concerted efforts to educate owners about meeting their cats' welfare needs so as to attenuate risks and improve cat physical and behavioral welfare outcomes.

## 1. Introduction

In the US, an estimated 74 to 85 million domestic cats (*Felis silvestris catus*) are thought to be kept as pets, making them the most commonly maintained companion animal [1, 2]. How we house, manage, and care for cats may impact owner attachment and cat health and behavior. These factors also influence outcomes such as whether cats remain in their homes or are ultimately abandoned or relinquished to shelters. An estimated 3.4 million cats of which approximately 1.4 million are euthanized are admitted to US animal shelters annually [3]. Behavioral reasons are among the most common causes for cat relinquishment. Based on a survey of cat owners, Salman et al. [4] reported that at least one behavioral reason was given for 28% of cat relinquishments. The most common included house-soiling (43.2%), problems between pets (18.9%), aggression toward people (14.6%), unfriendliness (5.4%), fearfulness (3.8%), and destructive behavior (12.4%). A significant association was found between relinquishment for such reasons and owning another household pet, suggesting that cats living in single animal households had a lower risk of relinquishment for behavioral reasons [5, 6]. Thus, the ways in which cats are housed (indoor or outdoor) and the social environments they experience may significantly impact their behavior, which in turn may have consequences for their well-being.

In regard to cat housing, it is now common practice for US veterinarians to advise clients to keep their cats indoors. The American Association of Feline Practitioners Statement on Confinement of Owned Indoor Cats-December 2007 (http://www.catvets.com/guidelines/position-statements/confinement-indoor-cats) takes the position that “Veterinarians are encouraged to educate clients and the public concerning the dangers associated with allowing cats' free-roam access to the outdoors… Additionally, these cats have an increased potential to be exposed to feline-specific and zoonotic diseases….”

Although indoor confinement reduces the risk of some health problems of cats, it also increases risk in other areas. For example, to optimize behavioral well-being, cats maintained indoors require attention to aspects of their environments that many owners may not be prepared to meet. According to the American Association of Feline Practitioners (AAFP) and the International Society of Feline Medicine (ISFM) recommendations for indoor-housed cats (referred to as the five pillars of a healthy feline environment) [7] include provision of (1) a safe place, (2) multiple and separated key environmental resources (food, water, toileting areas, scratching areas, play areas, and resting or sleeping areas), (3) opportunity for play and predatory behavior, (4) positive, consistent, and predictable human-cat social interaction, and (5) an environment that respects the importance of the cat's sense of smell (e.g., not removing scent marks, minimal use of harsh chemicals and perfumes, and use of synthetic pheromones).

In addition, indoor housing may be associated with certain physical health risks. Epidemiological studies have found that indoor housing is associated with increased risk (odds ratio) for a variety of common cat diseases, including odontoclastic resorptive lesions (~4.5), obesity (1.6–15.8), type 2 diabetes mellitus (1.4–4.6) [8], hyperthyroidism (4–11.2), and behavioral disorders [8, 9].

Given the popularity of the cat as an indoor-housed companion animal, understanding the extent to which owners provide care, management, and housing that meets their needs may help to promote their well-being and avoid adverse health and welfare outcomes for them. This pilot study therefore aimed to obtain information on the housing and management practices (including resources provided and feeding practices) used for companion cats by a cohort of veterinary professionals at two Midwestern veterinary colleges.

## 2. Materials and Methods

### 2.1. Survey

This data was collected as part of a larger project assessing cats' responses to environmental factors where cats were temporarily housed in cages for two or three days to simulate conditions and acclimation that might be experienced at a shelter [10]. The studies were conducted at The Ohio State University (OSU) and Purdue University (PU) Colleges of Veterinary Medicine. The questionnaire was intended to gather information on the housing environment, resources available to the cat, and frequency of certain behaviors including sickness and abnormal behaviors (the Appendix). Close-ended questions were used to identify cat signalment (age, breed, sex, neuter status, and declaw status), source of the cats (how they were obtained), housing environment, and feeding practices. Frequency of sickness and abnormal behaviors was reported on a scale of 0–5 where 0 = never, 1 = once, 2 = at least once/year, 3 = at least once/month, 4 = at least once/week, and 5 = at least once/day. Information about resources provided to cats in the home (e.g., toys and beds) was collected as yes, no, do not know, or not applicable.

### 2.2. Procedure

A sample of 130 cat-owner dyads (OSU, *N* = 74; PU, *N* = 56) from 81 households (OSU, *N* = 44; PU, *N* = 36) participated in the study. Owners were recruited from the faculty, staff, and students of The Ohio State University (OSU) and Purdue University (PU) veterinary colleges via email. Questionnaires were completed electronically. The protocols for the studies were reviewed and approved by the Institutional Review Board, the Animal Care and Use Committee, and the clinical trials committees of both The Ohio State University and Purdue University.

### 2.3. Statistical Analysis

Descriptive statistics were used and reported from each institution separately and as the pooled total. Analysis was performed using STATA IC 11 (StataCorp LP, College Station, TX, USA) statistical software.

## 3. Results

### 3.1. Signalment and Sourcing (Table 1)

All 130 cats in this study were neutered (female: *N* = 61; male: *N* = 69), 31% were declawed, and 95% were nonpedigreed. Ages ranged from 0.75 to 13 years (mean = 4.7 years). The average length of ownership was 4.0 years (range 0.3–12.75). The majority of cats were obtained from rescue or shelter (38%) or as a stray adult or kitten (44%). The remaining cats were obtained from friends (16%), were offspring of another owned cat (1%), or were gifts (1%). None were obtained from a breeder or pet store.

### 3.2. Housing Environment

The mean number of cats in the household was 2.1 with the maximum being 6 cats. The mean number of dogs in the household was 1.2 ranging from 0 to 8 dogs and the mean number of other animals in the house was 0.7 (SD 1.3) ranging from 0 to 7 animals (Table 2). Multicat households were more common than single-cat households (64% versus 36%). Of the multicat households, 81% consisted of unrelated individuals (OSU 84%; PU 80%).

Seventy-four percent of the cats were housed strictly indoors (OSU 84%; PU 61%) (Table 3). Sixty percent of households were attached or single houses with the remainder being apartments. The majority of owners (95%) fed diets consisting of 25% or less of wet food (Table 3).

The number of hours each day the owners spent in sight of their cat ranged from 1 to 24 (median 7.5). Time spent petting their cat each day ranged from 1 to 120 minutes (median 30) and reported time spent playing with their cat ranged from 0 to 90 minutes per day (median 20) (Table 4).

### 3.3. Sickness and Abnormal Behaviors

Owner-reported sickness and abnormal behaviors were dichotomized and reported as infrequent (once a year or less) or frequent (once a month or more often) (Table 5). The most commonly reported behaviors seen at least once per month were excessive appetite (34%), vomiting (25%), and nervous (22%) and aggressive (13%) behavior.

### 3.4. Resources

A summary of owner-reported resources is shown in Table 6. Owners reported that resting areas (31%), food/water bowls (53%), and litter boxes (30%) were located in areas where another animal could surprise them when they were in use. Twenty-one percent of cats did not have their own food bowl and 60% did not have their own water bowl. Thirty-five percent of cats did not have their own litter box in a private area of the house, 51% did not have litter boxes located in multiple areas of the house, and 73% were not washed regularly (at least monthly). Horizontal scratching opportunities were not provided to 38% of cats while vertical scratching opportunities were not provided to 22% of cats. Sixty percent of cats were not provided with chewing items such as cat grass, 32% were not provided toys to chase that mimic prey, and 78% did not have toys rotated to provide novelty.

## 4. Discussion

The results of this survey showed that, consistent with common US veterinary recommendations, most cats were confined to the indoors exclusively (74%); many lived in multicat households (64%), of which 81% lived with unrelated conspecifics. Ninety-five percent of the owned cats in this cohort were nonpedigreed and 98% had been acquired from a shelter, as a stray, or from a friend. None were purchased from a breeder or pet store which is less than has been previously reported (2% and 3%, resp.) [2]. Our respondents' cats were therefore primarily self-bred. This is not surprising given that of the “domesticated” species commonly kept in the US cats maintain the unique characteristic of owned populations interbreeding freely with feral populations rather than having mating strictly controlled by humans. Nonpedigree cats typically select their own mates and readily interbreed both with free-living feral domestic cats and with the wild progenitor species* F. silvestris,* where they coexist [11]. It is important to understand domestication when assessing the needs of cats. Evidence suggests that many of the differences between domestic and wild populations result from quantitative changes in the thresholds for performing a behavior rather than in qualitative changes in the behavior itself [12]. The cat's behavioral organization has been shaped by evolution to use information obtained from the environment to react to an event or to interact with an environmental feature to form rules of response for similar events or stimuli. The extent to which these “decision rules” of the ancestral species become altered by domestication may influence the negative subjective experiences (suffering) of an animal especially when there is a mismatch between an animal's current environment and the environment in which its decision rules evolved [13]. In this sense, we may consider domestic cats as similar to zoo animals, with the proximity of conspecifics and other animals, combined with limited resources and opportunities to express species-typical behavior potentially influencing cats' perceptions of control.

Additionally, cats have evolved as solitary hunters with typical social groups consisting of related females and their offspring. Adult males live on the periphery of a group of females with adolescent males, and often females, dispersing from their natal territory [14]. Yet the domestic cat, while less gregarious than other domestic species, does exhibit great plasticity in social behavior, with sociability appearing to be influenced by early experience and socialization [15]. While indoor living in itself and sharing of homes with other cats do not pose de facto feline welfare problems, living confined in close proximity to unrelated conspecifics may be stressful to some individual cats, particularly if they are not provided with adequate resources. Therefore, attention to the quality of the indoor housing environment is of critical importance to companion cat health and welfare.

Contrary to our expectations, however (given the owners' affiliations with veterinary medical colleges), the indoor housing environments provided to many of the cats were lacking in many of the resources most feline experts agree are essential for good welfare. At a minimum, these include individual resting, feeding, and elimination areas that are private and relatively free of unpredictable noise and interruption from other animals in the household. As noted previously, environmental enrichment that provides outlets for typical feline behaviors, such as novel toys that allow cats to “hunt,” daily play sessions with owners, scratching, and climbing opportunities, is also recommended to promote cat health and well-being [16]. Yet few owners reported routinely providing such resources to indoor-housed cats. Similar results have been reported for members of the general public elsewhere. For example, using owner self-reports to explore the living conditions of indoor-housed cats, Heidenberger [17] found that 24% of cats did not have their own food bowls and 51% had to share the litter pan with other cats.

An interesting finding was that 91% of cats were fed a diet consisting of 25% or less of wet food with 54% receiving no wet food at all. A review of the literature by Zoran and Buffington [18] found evidence suggesting factors relating to diet, including form (wet versus dry), composition (high carbohydrate versus high protein), and presentation (in a bowl or in a puzzle), are important environmental factors for domestic cats confined indoors [18]. Plantinga et al. [19] estimated the nutrient intake of feral cats based on data of food consumption patterns in order to understand the nutrient profile to which the cat has been evolutionarily adapted. The calculated diet was 69.5% water with daily energy intake from protein 52%, from fat 46%, and from NFE only 2%, supporting the fact that cats are truly obligate carnivores. In a pair of studies Hewson-Hughes et al. [20, 21] have indicated that providing wet food as well as dry may be beneficial to cat well-being. They demonstrated that cats will regulate their macronutrient intake to reach a “target” intake of total energy comprised of 52% protein, 36% fat, and 12% carbohydrate [20]. Importantly, this “target” could only be met by provision of wet foods in addition to dry and the cats consistently consumed more wet food (85% of total food intake) than dry when offered both [21]. More research is needed to determine if the shift from an obligatory meat-based natural diet to a meat-based and grain-based pet food rich in carbohydrates places the cat's metabolism under stress and possibly has unwanted negative health effects in the long run. Unfortunately, only limited conclusions can be drawn from this study as detailed information pertaining to diet was not collected which should be addressed in future studies.

Thirty-four percent of owners reported that their cats had excessive appetite at least once a month. This is in contrast to studies of cats housed in laboratories and in housing mimicking shelter environments, where* decreased* appetite was the most common abnormal behavior reported [10, 22, 23]. One explanation is that free-fed cats living in multicat households may have decreased appetite that goes unnoticed if changes in food intake are of short duration. Conversely, cats may use begging for food or overeating as an attention seeking behavior or in response to environmental stressors or negative emotional states [24].

A limitation of the current study is that body condition score was not recorded for the cats recruited from OSU. The mean body condition score of the cats recruited from PU (*N* = 56) was 6 ranging from 4 to 9 (using a 1–9 scale, 5 being ideal) indicating that the majority of cats were overweight or obese. It is important for future studies to assess if a correlation between excessive appetite and obesity exists. Obesity is now the most common nutritional disorder in cats in the United States. Studies in several countries have reported up to 63% of the cat population to be overweight or obese [25–29]. A recent report from the 2015 National Pet Obesity Awareness Day Survey conducted by the Association for Pet Obesity Prevention (APOP) noted that 58.2% or roughly 42 million cats in the US were found to be overweight or obese by their veterinarians [30]. Many factors contribute to this problem, including gender and hormonal changes due to neutering, age, inactivity, boredom due to indoor confinement, overfeeding, and feeding style (meal feeding versus free choice) [9, 18, 31]. Further, many cat health issues are associated with obesity, including type-2 diabetes mellitus, joint disease and lameness, development of feline lower urinary tract disease, idiopathic hepatic lipoidosis, and nonallergic skin condition [9]. Thus, the importance of owner attention to cats' diets, appetitive behaviors, and their implications for feline health cannot be overstated.

Another finding of particular interest was that owners reported that the most common sickness or abnormal behaviors in their cats were excessive appetite (34%), vomiting (25%), and nervous (22%) and aggressive (13%) behavior. Similar behaviors have been reported in other studies. For example, Morgan and Houpt [32] found the most common behavior problems to be scratching furniture (60%), eating houseplants (42%), conspecific aggression (36%), food stealing (25%), hissing/aggression to people (17%), house-soiling (16%), excessive vocalizations (16%), fabric chewing (7%), and “shyness” (4%). Later, Heidenberger [17] reported the most frequent behavior problems cited by cat owners to include anxiety (16.7%), scratching furniture (15.2%), feeding problems (10.9%), aggression (10.5%), and inappropriate urination (8.2%) and defecation in the house (5.1%). Many of these behaviors have been observed in response to inappropriate environments under laboratory conditions as well [22].

In summary, the current findings indicate that in our study population several owner oversights occurred relative to meeting best practices for indoor-housed cats as dictated by recent scientific findings and feline medicine practitioners' recommendations. However, the limitations of our study constrain drawing of broad conclusions, particularly as they apply to the general cat owning public. Cat-owner demographic information, such as age, level of education, and specific knowledge about cats and their physical and behavioral needs, was not obtained, which would be necessary to understand potential relationships between these factors and the housing and management practices adopted for cats. Additionally, owners' rationales for their housing and management choices were not investigated in this pilot study. Further, our sample is by no means representative of the US public, being limited to a very small pool of cat owners who were already participating in a larger study, which introduced sampling bias. Finally, validation studies of the survey tool should be conducted. Future studies should consider and address these factors to provide greater insight and more robust data.

It was anticipated that given their affiliation with veterinary medical schools this study population was likely to be familiar with recommended cat housing and management practices and, thus, would provide preliminary insight on how well best practices for meeting cats' needs were translated to and applied by those with relatively easy access to current information and education on the subject.

Nevertheless, the finding that faculty, staff, and students affiliated with both veterinary medical colleges did not appear to closely follow or implement many of the recommendations for optimal feline care suggests that there are significant gaps in translation of current findings about best cat care practices and their potential impacts on cat quality of life. Greater attention should be paid to developing more effective strategies for engaging and educating owners about meeting cats' comprehensive welfare needs.

## 5. Conclusion

Understanding cat owners' housing, care, and management practices is an important step toward promoting cat welfare. The results of this study indicate that even cat owners affiliated with veterinary medical colleges may not be fully aware of or implementing many best practices outlined for the welfare of cats housed indoors. This suggests a need for continuing education and “marketing” of messages about cats and their welfare needs so that all veterinary practitioners have the available tools, knowledge, and comfort to advise their cat owning clients. Ultimately, improved application and transfer of information to cat owners of current scientific information on the housing and handling is necessary to better meet cats' needs, promote the human-animal bond, and reduce risk of cat abandonment and relinquishment to shelters.

## Figures and Tables

**Table 1:** Cat signalment and demographic information of the subjects.

Characteristic	OSU (*N* = 74)		PU (*N* = 56)		Total (*N* = 130)	
	*N*	%	*N*	%	*N*	%
Gender						
Female	33	45	28	50	61	47
Male	41	55	28	50	69	53

Breed						
Nonpedigree	70	95	54	96	127	95
Other	4	5	2	4	7	5

Declawed						
Yes	17	23	23	41	40	31
No	57	77	33	59	90	69

Source						
Shelter/rescue	32	43	17	30	49	38
Stray/orphan	30	41	27	48	57	44
Friend	10	14	11	20	21	16
Offspring of owned cat	1	1	0	0	1	1
Gift	1	1	1	2	2	1
Breeder	0	0	0	0	0	0
Pet store	0	0	0	0	0	0

OSU = The Ohio State University; PU = Purdue University.

**Table 2:** Other animals in the home-the number of cats, dogs, and other pets in the home.

		OSU (*N* = 44)		PU (*N* = 36)		Total (*N* = 81)	
		*N*	%	*N*	%	*N*	%
*Cats*							
Single cat	1	13	30	16	44.5	29	36
Multicat	2	15	34	8	22	23	28
	3	7	16	9	25	16	20
	4	3	7	2	6	5	6
	5	0	0	0	0	0	0
	6	2	5	1	3	3	4

*Dogs *	0	20	45	9	25	29	36
	1	12	27	10	28	22	27
	2	6	14	12	33	18	22
	3	3	7	4	11	7	9
	4	2	5	1	3	3	4
	>5	1	2	0	0	1	1

*Other*	0	28	64	22	61	50	62
	1	7	16	5	14	12	15
	2	6	14	5	14	11	14
	>2	0	0	4	11	4	5

OSU = The Ohio State University; PU = Purdue University.

*N* = the number of cats and % = the percentage of the respondent population.

**Table 3:** Housing and diet.

	OSU (*N* = 74)		PU (*N* = 56)		Total (*N* = 130)	
	*N*	%	*N*	%	*N*	%
*Hours/day spent indoors*						
0–6	0	0	1	2	1	1
6–12	2	2.5	7	12	9	7
12–18	3	4	3	5	6	4
18–24	7	9.5	11	20	18	14
All	62	84	34	61	96	74

*% of diet fed as wet food*						
0	38	57	31	50	69	54
25	29	32	21	43	50	37
50	4	7	3	5	7	6
75	1	1	0	0	1	1
100	2	3	1	2	3	2

OSU = The Ohio State University; PU = Purdue University.

*N* = the number of cats and % = the percentage of the respondent population.

**Table 4:** Owner-cat interactions.

	OSU (*N* = 74)			PU (*N* = 56)			Total (*N* = 130)		
	Median	25% IQ	75% IQ	Median	25% IQ	75% IQ	Median	25% IQ	75% IQ
hr/day in sight of cat	7.5	5	12	5	2	6	6	5	10
min/day spent petting cat	30	20	49	30	15	39	30	15	40
min/day spent playing with cat	20	10	30	15	10	20	15	10	30

OSU = The Ohio State University; PU = Purdue University.

Median and 25th and 75th interquartile ranges are presented.

**Table 5:** Sickness and abnormal behavior.

	OSU		PU		Total		Total %
Behavior	IF	F	IF	F	IF	F	% F
Have excessive appetite	49	25	35	21	88	46	*34%*
Have little appetite	71	3	54	1	125	4	3%
Vomit (hair, food, and bile)	57	17	40	16	97	33	*25%*
Have diarrhea	73	1	55	1	128	2	2%
Have constipation	73	0	55	1	128	1	1%
Defecate outside of litter pan	70	4	52	4	122	8	6%
Strain/frequent attempts to urinate	73	1	56	0	129	1	1%
Urinate outside of litter pan	72	2	51	5	123	7	5%
Have blood in urine	74	0	55	0	129	0	0%
Spray urine	74	0	52	4	126	4	3%
Groom excessively	69	5	53	3	122	8	6%
Have excessive hair loss	73	1	54	2	127	3	2%
Scratch themselves excessively	72	2	54	2	126	4	3%
Have discharge from the eyes	64	10	53	3	117	13	10%
Seem nervous (anxious, fearful)	57	17	44	12	101	29	*22%*
Seem aggressive	64	10	48	7	112	17	*13%*

OSU = The Ohio State University; PU = Purdue University.

IF = infrequent, once a year or less; F = frequent, monthly or more often.

**Table 6:** Owner-reported resources provided to cats (%).

	OSU (*N* = 74)				Purdue (*N* = 56)				Total (*N* = 130)			
	No	Yes	DK	N/A	No	Yes	DK	N/A	No	Yes	DK	N/A
Does each cat have its own resting area in a convenient location that provides some privacy?	8	91	1	0	5	92	0	3	7	92	1	2
Are resting areas located such that another animal cannot sneak up on the cat while it rests?	34	65	1	0	22	61	6	11	28	63	4	6
Are resting areas located away from appliances or air ducts (machinery) that could come on unexpectedly while the cat rests?	7	91	1	1	11	89	0	0	9	90	1	1
Does each cat have the opportunity to move to a warmer or cooler area if it chooses to?	3	96	1	0	0	100	0	0	2	98	1	0
Does each cat have its own food bowl?	19	80	1	0	23	74	0	3	21	77	1	2
Does each cat have its own water bowl?	58	39	1	1	58	39	0	3	58	39	1	2
Are the bowls located in a convenient location that provides some privacy while it eats or drinks?	19	76	5	0	17	83	0	0	18	80	3	0
Are bowls located such that another animal cannot sneak up on this cat while it eats or drinks?	49	45	4	3	47	39	3	11	48	42	4	7
Are bowls located away from machinery that could come on unexpectedly?	11	88	1	0	14	86	0	0	13	87	1	0
Does each cat have its own box in a convenient, well-ventilated location that still gives the cat some privacy while using it (1 litter box per cat + 1)?	39	60	1	0	28	64	2	6	34	62	2	3
Are boxes located in more than one area of the house?	30	34	3	34	47	39	3	11	39	37	3	23
Are boxes located so another animal cannot sneak up on the cat during use?	31	62	3	4	22	64	0	14	27	63	2	9
Are boxes located away from machinery that could come on unexpectedly during use?	14	85	1	0	14	83	3	0	14	84	2	0
Are boxes washed regularly (at least monthly) with a mild detergent (like dishwashing liquid), rather than strongly scented cleaners?	68	32	0	0	75	19	3	3	72	26	2	2
Does each cat have the opportunity to engage in play with other animals or the owner if it chooses to on a daily basis?	1	96	3	0	5	92	0	3	3	94	2	2
Does each cat have the option to disengage from other animals or people in the household at all times?	8	90	1	0	3	94	0	3	6	92	1	2
Are horizontal scratching posts provided?	20	78	1	0	56	44	0	0	38	61	1	0
Are vertical scratching posts provided?	8	92	0	0	36	64	0	0	22	78	0	0
Are chew items (e.g., cat-safe grasses) provided?	49	49	3	0	67	28	5	0	58	39	4	0
Does each cat have toys to chase that mimic quickly moving prey?	32	67	0	1	31	69	0	0	32	68	0	1
Does each cat have toys that can be picked up, carried, and tossed in the air?	4	96	0	0	11	89	0	0	8	93	0	0
Are toys rotated on a regular basis (at least weekly) to provide novelty?	68	32	0	0	86	11	3	0	77	22	2	0

OSU = The Ohio State University; PU = Purdue University.

DK = do not know; N/A = not applicable.

## Appendix

### Questionnaire (Adapted from [33])

*Cat and Client History Form*

Owner name: —
Cat's name: —
Date: —
Contact Information:
  Phone #: —
  E-mail: —
Breed: —
Date of Birth: —
Weight: — lb/kg
Sex: (circle one)
  FI
  FS
  MI
  MN
Declawed?
  No: —
  Yes: —
  If yes,
    Front: —
    All: —
Owned How Long?
  — Years, — months
Total Cats: —
Total Dogs: —
Other Pets: —
Other people: —
Housing: Apartment:
  ⬜ studio,
  ⬜ 1-2 bedrooms,
  ⬜ 3 or more bedrooms,
  ⬜ attached house/twin duplex,
  ⬜ attached house, 3 or more units,
  ⬜ single house,
  ⬜ other —
Previous Illnesses or Surgeries: —

*Directions*. For items below, please use the following choices to describe how many times you have seen your pet experience the symptom, adding* comments/explanation* **– **as appropriate* Score *=

0 = I have* NEVER* seen it
1 = I have seen it at least* ONCE*
2 = I see it at least* ONCE per YEAR*
3 = I see it at least* ONCE per MONTH*
4 = I see it at least* ONCE per WEEK*
5 = I see it* DAILY*

How often does your cat:

Have excessive appetite
  *Score:* —
  *Comments/explanation:* —
Have little appetite
  *Score:* —
  *Comments/explanation:* —
Vomit (food, hair, bile, other)
  *Score:* —
  *Comments/explanation:* —
Have diarrhea
  *Score:* —
  *Comments/explanation:* —
Have constipation
  *Score:* —
  *Comments/explanation:* —
Defecate outside the litter box
  *Score:* —
  *Comments/explanation:* —
Strain or have frequent attempts to urinate
  *Score:* —
  *Comments/explanation:* —
Urinate outside the litter box
  *Score:* —
  *Comments/explanation:* —
Have blood in the urine
  *Score:* —
  *Comments/explanation:* —
Spray urine
  *Score:* —
  *Comments/explanation:* —
Grooms excessively
  *Score:* —
  *Comments/explanation:* —
Have excessive hair loss
  *Score:* —
  *Comments/explanation:* —
Scratch excessively
  *Score:* —
  *Comments/explanation:* —
Have discharge from eyes
  *Score:* —
  *Comments/explanation:* —
Seem nervous (anxious)
  *Score:* —
  *Comments/explanation:* —
Seem fearful
  *Score:* —
  *Comments/explanation:* —
Seem Aggressive
  *Score:* —
  *Comments/explanation:* —
Seem “needy” of contact or attention
  *Score:* —
  *Comments/explanation:* —

Please check the box that best applies to your cat

Diet: wet food (name —)
  ⬜ None
  ⬜ 25%
  ⬜ 50%
  ⬜ 75%
  ⬜ 100%
Diet: dry food (name —)
  ⬜ None
  ⬜ 25%
  ⬜ 50%
  ⬜ 75%
  ⬜ 100%
Litter type: (clumping, clay, recycled paper, etc)
  —
How many hours each day, on average, does your cat spend indoors? (check one)
  ⬜ 0–6
  ⬜ 6–12
  ⬜ 12–18
  ⬜ 18–24
  ⬜ Indoor Only
If you have more than one cat, what is their relationship?
  ⬜ Not Related
  ⬜ Littermate
  ⬜ Sibling
  ⬜ Parent-Offspring
  ⬜ Single Cat Household
  ⬜ Other
Where did you obtain your cat (source)?
  ⬜ Shelter
  ⬜ Offspring from a pet I already own(ed)
  ⬜ Purchased from a friend
  ⬜ Purchased from a breeder
  ⬜ Purchased from a pet shop
  ⬜ Gift
  ⬜ Stray/orphan
  ⬜ Other

*Client Resource Checklist*. The following questions ask about your cat's resources because we want to learn more about your cat's environment. Please ✓ DK if you don't know, NA if a question does not apply to your home, or Yes or No after each question.

*Space*

(1) Does each cat have its own resting area in a convenient location that provides some privacy?
  ⬜ DK
  ⬜ NA
  ⬜ Yes
  ⬜ No
  Other/Comments: —
(2) Are resting areas are located such that another animal cannot sneak up on the cat while it rests?
  ⬜ DK
  ⬜ NA
  ⬜ Yes
  ⬜ No
  Other/Comments: —
(3) Are resting areas are located away from appliances or air ducts (machinery) that could come on unexpectedly while the cat rests?
  ⬜ DK
  ⬜ NA
  ⬜ Yes
  ⬜ No
  Other/Comments: —
(7) Does each cat have the opportunity to move to a warmer or cooler area if it chooses to?
  ⬜ DK
  ⬜ NA
  ⬜ Yes
  ⬜ No
  Other/Comments: —
(8) Is a radio or TV left playing when the cat is home alone?
  ⬜ DK
  ⬜ NA
  ⬜ Yes
  ⬜ No
  Other/Comments: —

*Food and Water*

(9) Does each cat have its own food bowl?
  ⬜ DK
  ⬜ NA
  ⬜ Yes
  ⬜ No
  Other/Comments: —
(10) Does each cat have its own water bowl?
  ⬜ DK
  ⬜ NA
  ⬜ Yes
  ⬜ No
  Other/Comments: —
(11) Are the bowls located in a convenient location that provides some privacy while it eats or drinks?
  ⬜ DK
  ⬜ NA
  ⬜ Yes
  ⬜ No
  Other/Comments: —
(12) Are bowls located such that another animal cannot sneak upon this cat while it eats or drinks?
  ⬜ DK
  ⬜ NA
  ⬜ Yes
  ⬜ No
  Other/Comments: —
(14) Are bowls located away from machinery that could come on unexpectedly?
  ⬜ DK
  ⬜ NA
  ⬜ Yes
  ⬜ No
  Other/Comments: —

*Litter Boxes*

(15) Does each cat have its own box in a convenient, well-ventilated location that still gives the cat some privacy while using it (1 litter box per cat + 1)?
  ⬜ DK
  ⬜ NA
  ⬜ Yes
  ⬜ No
  Other/Comments: —
(16) Are boxes located in more than one area of the house?
  ⬜ DK
  ⬜ NA
  ⬜ Yes
  ⬜ No
  Other/Comments: —
(17) Are boxes located so another animal cannot sneak up on the cat during use?
  ⬜ DK
  ⬜ NA
  ⬜ Yes
  ⬜ No
  Other/Comments: —
(18) Are boxes located away from machinery that could come on unexpectedly during use?
  ⬜ DK
  ⬜ NA
  ⬜ Yes
  ⬜ No
  Other/Comments: —
(20) Are boxes washed regularly (at least monthly) with a mild detergent (like dishwashing liquid), rather than strongly scented cleaners?
  ⬜ DK
  ⬜ NA
  ⬜ Yes
  ⬜ No
  Other/Comments: —
(22) Is the brand or type of litter purchased changed infrequently (less than monthly)?
  ⬜ DK
  ⬜ NA
  ⬜ Yes
  ⬜ No
  Other/Comments: —

*Social Contact*

(24) Does each cat have the opportunity to engage in play with other animals or the owner if it chooses to on a daily basis?
  ⬜ DK
  ⬜ NA
  ⬜ Yes
  ⬜ No
  Other/Comments: —
(25) Does each cat have the option to disengage from other animals or people in the household at all times?
  ⬜ DK
  ⬜ NA
  ⬜ Yes
  ⬜ No
  Other/Comments: —
(26) Do any cats interact with outdoor cats through windows?
  ⬜ DK
  ⬜ NA
  ⬜ Yes
  ⬜ No
  Other/Comments: —
(27) How many hours a day are you in sight of your cat?
  ⬜ DK
  — (h/day)
  Other/Comments: —
(28) How many minutes a do you spend petting your cat?
  ⬜ DK
  — (min/day)
  Other/Comments: —
(29) How many minutes a do you spend playing with your cat?
  ⬜ DK
  — (min/day)
  Other/Comments: —

*Body Care and Activity*

(30) Are horizontal scratching posts provided?
  ⬜ DK
  ⬜ NA
  ⬜ Yes
  ⬜ No
  Other/Comments: —
(31) Are vertical scratching posts provided?
  ⬜ DK
  ⬜ NA
  ⬜ Yes
  ⬜ No
  Other/Comments: —
(32) Are chew items (e.g., cat-safe grasses) provided?
  ⬜ DK
  ⬜ NA
  ⬜ Yes
  ⬜ No
  Other/Comments: —
(33) Does each cat like to play with toys?
  ⬜ DK
  ⬜ NA
  ⬜ Yes
  ⬜ No
  Other/Comments: —
(34) Does each cat have toys to chase that mimic quickly moving prey?
  ⬜ DK
  ⬜ NA
  ⬜ Yes
  ⬜ No
  Other/Comments: —
(35) Does each cat have toys that can be picked up, carried, and tossed in the air?
  ⬜ DK
  ⬜ NA
  ⬜ Yes
  ⬜ No
  Other/Comments: —
(36) Are toys rotated on a regular basis (at least weekly) to provide novelty?
  ⬜ DK
  ⬜ NA
  ⬜ Yes
  ⬜ No
  Other/Comments: —
